# Biofilm formation at the solid-liquid and air-liquid interfaces by *Acinetobacter *species

**DOI:** 10.1186/1756-0500-4-5

**Published:** 2011-01-11

**Authors:** Sara Martí, Jesús Rodríguez-Baño, Manuella Catel-Ferreira, Thierry Jouenne, Jordi Vila, Harald Seifert, Emmanuelle Dé

**Affiliations:** 1 Laboratory "Polymères, Biopolymères, Surfaces", University of Rouen, UMR 6270 & FR 3038 CNRS, IFRMP23, Mont-Saint-Aignan, France; 2 Infectious Diseases and Clinical Microbiology Unit, Hospital Universitario Virgen Macarena, Seville, Spain; 3 Department of Microbiology, Hospital Clinic, Barcelona, Spain; 4 Institute for Medical Microbiology, Immunology and Hygiene, University of Cologne, Cologne, Germany

## Abstract

**Background:**

The members of the genus *Acinetobacter *are Gram-negative cocobacilli that are frequently found in the environment but also in the hospital setting where they have been associated with outbreaks of nosocomial infections. Among them, *Acinetobacter baumannii *has emerged as the most common pathogenic species involved in hospital-acquired infections. One reason for this emergence may be its persistence in the hospital wards, in particular in the intensive care unit; this persistence could be partially explained by the capacity of these microorganisms to form biofilm. Therefore, our main objective was to study the prevalence of the two main types of biofilm formed by the most relevant *Acinetobacter *species, comparing biofilm formation between the different species.

**Findings:**

Biofilm formation at the air-liquid and solid-liquid interfaces was investigated in different *Acinetobacter *spp. and it appeared to be generally more important at 25°C than at 37°C. The biofilm formation at the solid-liquid interface by the members of the ACB-complex was at least 3 times higher than the other species (80-91% versus 5-24%). In addition, only the isolates belonging to this complex were able to form biofilm at the air-liquid interface; between 9% and 36% of the tested isolates formed this type of pellicle. Finally, within the ACB-complex, the biofilm formed at the air-liquid interface was almost 4 times higher for *A. baumannii *and *Acinetobacter *G13TU than for *Acinetobacter *G3 (36%, 27% & 9% respectively).

**Conclusions:**

Overall, this study has shown the capacity of the *Acinetobacter *spp to form two different types of biofilm: solid-liquid and air-liquid interfaces. This ability was generally higher at 25°C which might contribute to their persistence in the inanimate hospital environment. Our work has also demonstrated for the first time the ability of the members of the ACB-complex to form biofilm at the air-liquid interface, a feature that was not observed in other *Acinetobacter *species.

## Findings

The observation of natural habitats has shown that bacteria generally aggregate in biofilm structures to persist [1]. Biofilm is an association of microbial cells which are surrounded by a matrix of polysaccharide material; this structure is an optimal environment for genetic material exchange between the different microorganisms [2]. Biofilm formation has been linked to the survival of pathogenic bacteria in the hospital environment and it has been connected to infections associated with indwelling medical devices. Indeed, biofilm bacterial communities confer a protection from environmental hazards [1,3]. The surface colonization can take place either at the solid-liquid interface (SLI-biofilm) or at the air-liquid interface (ALI-biofilm) where it forms a pellicle on the top of the liquid media as observed in other microorganisms such as *Pseudomonas aeruginosa *or *Salmonella *spp. [4,5].

The members of the genus *Acinetobacter *are ubiquitous Gram-negative cocobacilli that are frequently found in the environment but also in the hospital setting where they have been associated with outbreaks of nosocomial infections [6]. The so-called *Acinetobacter calcoaceticus - Acinetobacter baumannii *(ACB) complex contains phenotypically closely related species, i.e. the clinically most important species, *A. baumannii, Acinetobacter *Genospecies 3 and *Acinetobacter *Genospecies 13TU, also referred to as the *A. baumanni *group, and the environmental species *A. calcoaceticus *[7]. Although these species are very difficult to differentiate in the laboratory, their importance in the clinical environment is clearly different: *A. baumannii *and *Acinetobacter *Genospecies 13TU are responsible for most of the infections while *Acinetobacter *Genospecies 3 is less often associated with disease. On the other hand, *A. calcoaceticus *is mainly an environmental microorganism rarely involved in human infections [7]. Among them, *A. baumannii *has emerged as the most common pathogenic species involved in hospital-acquired infections [6,8-10]; this multiresistant opportunistic pathogen can survive on nutrient-limited surfaces for several days, even in dry conditions and in the harsh hospital environment [11]. One reason for this emergence may be its persistence in the hospital wards, in particular in the intensive care unit; this persistence could be partially explained by the capacity of these microorganisms to form biofilm. Therefore, our main objective was to study the prevalence of the two main types of biofilm formed by the most relevant *Acinetobacter *species. Overall, this study has shown the capacity of the members of the ACB-complex to form two different types of biofilm (SLI and ALI), and it revealed that biofilm formation increased at 25°C, a condition that might contribute to their persistence in the hospital environment.

### Bacterial strains

This study has investigated the ALI and SLI-biofilm formation in different *Acinetobacter *spp.: 64 clonally unrelated *A. baumannii *clinical isolates collected during the GEIH-Ab2000 project [12]; *Acinetobacter johnsonii *(n = 34); *Acinetobacter lwoffii *(n = 26); *Acinetobacter radioresistens *(n = 20); *Acinetobacter *Genospecies 3 (n = 46); *Acinetobacter *Genospecies 13TU (n = 60); *A. calcoaceticus *(n = 10); *Acinetobacter junii *(n = 5). The *Acinetobacter *spp. other than *A. baumannii *were mainly recovered from catheter-related bloodstream infections and from the skin of patients outside the ICU and of healthy controls [13-15]. Both biofilm analyses were carried out on all the isolates in parallel.

### SLI-Biofilm formation

SLI-biofilm formation was performed in 96-well plates; biofilm formation was determined in Mueller Hinton broth (Oxoid, France) using an initial OD_600 _of 0.01 and incubated at 25°C or 37°C for 48 h without shaking. Two wells were left uninoculated and used as negative controls. After checking that all the isolates had grown at a similar rate, the culture media was removed by inversion and the wells were washed twice with distilled water. The biofilm was stained with 0.5% crystal violet (w/v) for 20 minutes at room temperature and the wells were washed again to remove the unbound crystal violet. Biofilm formation was finally quantified at 550 nm after solubilisation with 95% ethanol. The bacterial isolates were considered to be positive for SLI-biofilm formation when the readings obtained were at least 3 times greater than the negative control.

### ALI-Biofilm formation

**Figure 1:**
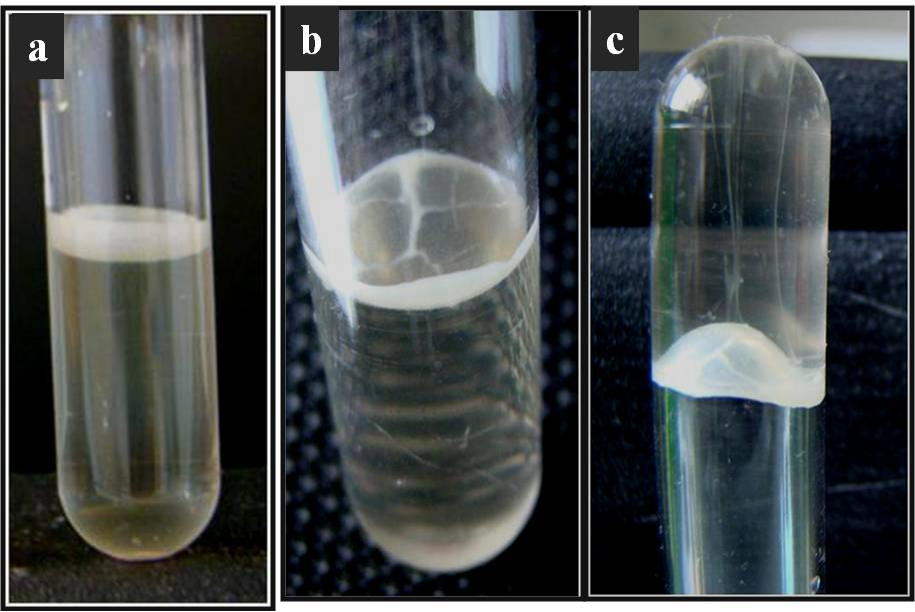
**ALI-biofilm formation by *A. baumannii***. a) the positive strain forms a pellicle on the top of the liquid media and the culture broth remains transparent; b) pellicle observed from above; c) inversed tube shows the pellicle strength.

ALI-biofilm formation was performed in 5 ml polystyrene tubes with a diameter of 12.8 mm; biofilm formation was determined in Mueller Hinton broth (Oxoid, France) using an initial OD_600 _of 0.01 and incubated at 25°C or 37°C for 72 h without shaking. Positive ALI-biofilm samples were identified visually (Figure 1); the isolates were considered positive when a pellicle was covering the whole liquid surface.

### Data analysis

All the experiments were performed in duplicate at two independent time-points. The percentages of isolates producing biofilm were compared by the chi squared test, or the Fisher exact test, as appropriate. A 2- tailed P value < 0.05 was considered significant. The percentage of isolates of the various *Acinetobacter *spp. producing biofilm is illustrated in Table 1.

**Table 1:** SLI and ALI-biofilm formation in *Acinetobacter *spp.

	ALI-Biofilm		SLI-Biofilm	
	
	25°C	37°C	25°C	37°C
***A. baumannii *(n = 64)**	23 (35.9)	11 (12.2)	52 (81.3)	(16)^a^
***Acinetobacter *G3 (n = 46)**	4 (8.7)	1 (2.2)	42 (91)	42 (91)
***Acinetobacter *G13TU (n = 60)**	16 (26.7)	9 (15)	48 (80)	34 (56.7)
***A. johnsonii *(n = 34)**	0	0	8 (23.5)	5 (14.7)
***A. lwoffii *(n = 26)**	0	0	3 (11.5)	3 (11.5)
***A. radioresistens *(n = 20)**	0	0	1 (5)	3 (15)
***A. calcoaceticus *(n = 10)**	3 (30)	0	7 (70)	2 (20)
***A. junii *(n = 5)**	3 (60)	3 (60)	3 (60)	2 (40)

Data are expressed as number (percentage) of isolates

^a ^The percentage of SLI-Biofilm formation in this collection of *A. baumannii *clinical isolates has already been published by Rodriguez-Baño *et al *(16); this study showed that 63% of the clinical isolates were forming SLI-biofilm at 37°C

## Results and discussion

As shown in Table 1 SLI-biofilm was more frequently produced at both 25°C and 37°C in *Acinetobacter *Genospecies 3, *Acinetobacter *Genospecies 13TU, and *A. baumannii *than in *A. johnsonii*, *A. lwoffii*, and *A. radioresistens *(p < 0.05 for all comparisons). Thus, these results might explain, at least in part, the marked persistence of the three former species in hospitals and their involvement in nosocomial infections. Moreover, in a previous study (using the same collection of *A. baumannii *clinical isolates), Rodriguez-Baño *et al. *[16] showed that 63% of *A. baumannii *clinical isolates formed SLI-biofilm at 37°C. Based on these results, our study revealed that the rates of SLI-biofilm formation obtained for *Acinetobacter *Genospecies 13TU and *A. baumannii *were similar, as well as their variation related to the temperature (higher at 25°C than at 37°C). Likewise, ALI-biofilm was significantly more frequent at both 25°C and 37°C in *Acinetobacter *Genospecies 13TU and *A. baumannii *than in *A. johnsonii*, *A. lwoffii*, *A. radioresistens*, and *Acinetobacter *Genospecies 3 (p < 0.05). Overall, this similar behaviour in biofilm formation between *A. baumannii *and *Acinetobacter *G13TU is coherent with the fact that these two species are the most commonly found in the hospital.

Our results showed (see Table 1) that ALI-biofilm was mainly formed by the members of the ACB-complex. Although *A. junii *had some capacity to form this characteristic biofilm, the number of isolates studied was too small to draw conclusions. By contrast, as a member of the ACB-complex, *A. calcoaceticus *has shown a different pattern at 37°C with a complete absence of ALI-biofilm formation together with a reduced ability to form SLI-biofilm, both of which could be explained by the fact that this species' natural habitat is the environment where lower temperatures usually prevail.

Finally, as biofilm formation generally predominated at 25°C rather than 37°C, this mechanism could explain the observed persistence of the members of the *A. baumannii *group in the inanimate hospital environment. Previous studies have already reported that the members of the *A. baumannii *group and especially *A. baumannii *survive desiccation better than other *Acinetobacter *spp., comparing the survival rate of *A. baumannii *to those obtained for *Staphylococcus aureus *[7,17]. In addition, in a recent study, Wisplinghoff *et al *showed that all disinfectants tested inhibited the growth of *A. baumannii *[18] which suggests that these microorganisms in their planktonic state are susceptible to most disinfectants. As already demonstrated for other bacterial species, extracellular polymeric substances from the biofilm matrix play an important role in the resistance and tolerance to dehydration [19], suggesting that biofilm formation contributes to the ability of *A. baumannii*, and possibly the other members of the *A. baumannii *group, to survive better in the hospital environment and to resist the action of disinfectants.

For *A. baumannii*, SLI-biofilm has already been described in several reports [20-22] and it has been linked to some device-associated infections [16]. Indeed, de Breij *et al *[21] have recently compared SLI-biofilm formation by the members of the ACB complex and concluded that there was no difference between clinically relevant and less-relevant *Acinetobacter *strains and species; in the same way, no temperature related differences were found for biofilm formation. Our results slightly differ from theirs possibly due to the higher number of strains and species analysed. They suggested a reduced biofilm formation for the *Acinetobacter *Genospecies 13TU although only 3 isolates had been studied; by contrast, after studying 60 isolates, our results clearly show that the behaviour for biofilm formation of this species is highly related to *A. baumannii*. On the other hand, although we have only studied 10 *A. calcoaceticus *isolates, the results also indicate an association between biofilm and temperature in this non-pathogenic species, a tendency that can be observed in most of the analysed species. The comparison with other non-pathogenic species outside the ACB-complex has shown a relevant difference for biofilm formation that was even increased when looking at the ALI-biofilm formation, an ability that to our knowledge has never been described in clinical isolates of the *Acinetobacter *spp.

In summary, the members of the *A. baumannii *group have a higher ability to form SLI-and ALI-biofilm than other less clinically related species. Nevertheless, *A. calcoaceticus*, the environmental representative of the ACB-complex, showed a reduced biofilm formation at 37°C when compared to the other members of this complex. This feature could be connected to the higher colonization rate of patients by pathogenic *Acinetobacter *species (mainly *A. baumannii *and *Acinetobacter *Genospecie 13TU), and probably contributing to the increased risk of clinical infection.

## List of abbreviations

SLI: Solid-Liquid Interface; ALI: Air-Liquid Interface; ACB-Complex: *Acinetobacter calcoaceticus - Acinetobacter baumannii *complex

## Competing interests

The authors declare that they have no competing interests.

## Authors' contributions

SM carried out the biofilm studies, participated in the design of the study and drafted the manuscript; JRB performed the statistical analysis and helped to draft the manuscript; MCF helped to perform the biofilm studies; TJ participated in the design of the study; JV participated in the design of the study and helped to draft the manuscript; HS participated in the design of the study and helped to draft the manuscript; ED participated in the design and coordination of the study and helped to draft the manuscript. All authors have read and approved the final manuscript.

## Consent

Due to the observational and retrospective design of the study, the Ethic Committee of the participating centres waived the need for obtaining written informed consent.
